# Greater postprandial glucose excursions and inadequate nutrient intake in youth with type 1 diabetes and celiac disease

**DOI:** 10.1038/srep45286

**Published:** 2017-03-24

**Authors:** Anna Pham-Short, Kim C Donaghue, Geoffrey Ambler, Sarah Garnett, Maria E. Craig

**Affiliations:** 1 Institute of Endocrinology and Diabetes, The Children’s Hospital at Westmead, Sydney, Australia; 2 Discipline of Child and Adolescent Health, University of Sydney, Sydney, Australia; 3 School of Women’s and Child’s Health, University of New South Wales, Sydney, Australia

## Abstract

The gluten free diet (GFD) has a high glycemic index and low-fiber content, which potentially influences glycemic excursions in type 1 diabetes (T1D) and celiac disease (CD). Participants in this case-control study of youth with T1D+CD (n = 10) and T1D only (n = 7) wore blinded continuous glucose monitoring systems for six days. Blood glucose levels (BGLs) were compared between groups for each meal, including pre-meal, peak, 2-hour postprandial and time-to-peak. Participants consumed a test-breakfast of GF cereal and milk for three days and kept weighed food diaries; nutrient intake was analyzed and compared to national recommendations. Youth with T1D+CD had shorter time-to-peak BGL (77 vs 89 mins, *P* = 0.03), higher peak (9.3 vs 7.3 mmol/L, *P* = 0.001) and higher postprandial BGLs than T1D (8.4 vs 7.0 mmol/L, *P* = 0.01), despite similar pre-meal BGLs (9.2 vs 8.6 mmol/L, *P* = 0.28). Regarding test breakfast, greater pre and post-meal BGL difference correlated with longer CD duration (R = 0.53, *P* = 0.01). Total energy and macronutrient intake didn’t differ between groups; however the majority of participants collectively had inadequate intake of calcium (76%), folate (71%) and fiber (53%), with excessive saturated fat (12%) and sodium (>2,000 mg/day). The GFD is associated with greater glycemic excursions and inadequate nutritional intake in youth with T1D+CD. Clinical management should address both glycemic variability and dietary quality.

The gluten free diet (GFD) recommended for management of celiac disease (CD) requires the complete avoidance of wheat, rye and barley, which is often replaced by white rice or corn^1^. Such foods may contribute to a higher glycemic index^2^, higher glycemic load^3^ and lower fiber diet^4^,^5^ compared to gluten containing equivalents. The well-known benefits of a higher fiber diet in the general population include cardiovascular, and bowel health^6^ and in type 1 diabetes (T1D), these benefits extend to improved glycemic control^7^.

Restrictive diets such as the GFD are at risk of nutritional deficiencies, requiring increased vigilance in growing children and adolescents^8^. The mandatory fortification of wheat flour with micronutrients such as thiamin and folic acid^9^, but not gluten-free grains further places those consuming a GFD at risk of inadequate nutrient intake. Studies investigating the nutritional composition of the GFD in adults have shown inadequacies of specific micronutrients including thiamin, folate, vitamin A and calcium^5^,^10^. However there are limited dietary studies in youth with CD^4^,^11^,^12^ and none in children with co-existing T1D.

Adults and youth with CD have a lower carbohydrate intake compared with the general population^5^,^12^, which has been attributed to poor palatability and increased costs of the GFD^13^. Carbohydrate intake in children with T1D is often at the lower level of dietary guidelines^14^, which may be due to a conscious effort to reduce the risk of postprandial hyperglycemia^15^. In studies of children with CD, fiber and iron intakes are also significantly lower than recommendations^12^,^16^, due to the naturally low fiber and iron content of GF grains^10^. However, whether the coexistence of T1D+CD is associated with a greater reduction in carbohydrate and fiber intake has not been previously examined. It is also unknown whether the traditional GFD, with a higher glycemic index and lower fiber content, impacts on glycemic variability in this population. Therefore the aims of this study were to compare, (i) post-prandial excursions (PPE) and (ii) macronutrient, micronutrient and fiber intake in youth with T1D+CD compared to those T1D alone. We hypothesized that youth with T1D+CD would demonstrate greater glycemic excursions and lower nutrient intake.

## Research Design and Methods

### Subjects

This was a case-control, observational study of youth with T1D and biopsy-proven CD, individually matched to controls with T1D only by age, diabetes duration and HbA1c ( ± 0.5%, 3 mmol/mol). Inclusion criteria were T1D+CD, age <18 years, treatment with either multiple daily injections or insulin pump therapy, willingness to test at least four blood glucose levels (BGLs) per day, and the ability to read and record food diaries in the English language. Participants were recruited from the Diabetes Clinic at The Children’s Hospital at Westmead, a tertiary pediatric hospital in Sydney, Australia. We aimed to recruit 10 youth with T1D+CD and 10 with T1D. The sample size was based on a minimum mean post-prandial BGL difference of 3 mmol/L (standard deviation 3 mmol/L) between groups over three days (30 meals per group), with α = 0.5 and power 80%, assuming a drop out or missed BGL rate of 20%. The study protocol included youth wearing a blinded continuous glucose monitoring system (CGMS), consumption of the same test breakfast for three days, completion of a weighed food diary and documentation of diabetes care (injections or pumps, daily insulin doses and all BGLs measured using a glucometer). All participants attended their diabetes review appointment with their pediatric endocrinologist in the six weeks prior to the study visit for adjustment of insulin doses, or insulin pump settings. Participants used their pre-exiting insulin to carbohydrate ratios and insulin sensitivity factors, which had been set and adjusted according to standard clinical criteria^17^.

The study was approved by the Sydney Children’s Hospital Network Human Research Ethics Committee (study number: 12/SCHN/21, registration date 7^th^ November 2012). The methods were carried out in accordance with the relevant guidelines and regulations. Informed consent was obtained from parents and assent from children aged 12–16 years prior to participation.

### Study visit

All participants were assessed by an Accredited Practising Dietitian (A.P-S.). Patients underwent a structured interview, which involved documentation of their typical daily food intake, including weekend variations for meals both inside and outside the home. Carbohydrate counting skills were reviewed using food replicas (Mentone Educational, Australia), food packaging and real food (such as breakfast cereal). Measuring scales and cups were used as educational tools to assist with quantifying carbohydrate intake. Anthropometric measures were taken with the participant in light clothing and without shoes. Height was measured to the nearest 0.1 cm (Harpenden stadiometer) and weight was measured to the nearest 0.1 kg; BMI was calculated with z-scores computed using Centers for Disease Control and Prevention (CDC) 2000 reference data.

### Continuous Glucose Monitoring System

CGMS (Medtronic Enlite Sensor with a blinded iPro2 transmitter) was inserted by the diabetes educator and worn uninterrupted for six days. Details of the sensor properties are described in detail elsewhere^18^. Patients were asked to perform at least four BGLs per day; prior to main meals and before bed with their own glucometers. Glycemic targets were set as 4.0–7.8 mmol/l, with hypoglycemia defined as a blood glucose level (BGL) ≤ 3.9 mmol/mol, euglycemia between 4.0–7.8 mmol/mol, and hyperglycemia as ≥7.9 mmol/mol.

### CGMS Data Analysis

Participants were stratified by the presence or absence of CD co-existence for data analysis. For each main meal consumed and accompanied by a pre-prandial insulin bolus, time to reach peak BGL, peak BGL, and 2 hour postprandial BGL were examined. The following variability parameters were measured:

- Total variability (SD_T_) was calculated as the standard deviation (SD) for all of the measurements for all of the study days^19^.
- Within day glucose variability (SD_w_) was calculated as the SD of all measurements in a 24 hour period, averaged over the sensor reading days^19^.
- Between day variability was measured by Mean of daily differences (MODD), and calculated as the average of the absolute difference in glucose values at the exact time of day (midnight), and averaged over five days^19^.
- Meal effect was measured by Area Under the Curve (AUC) and calculated as the sum of the absolute value of excursions from sensor value at the start of the meal and was calculated for 2 hours^20^ following the start of the meal.

### Dietary intake

Participants maintained a standardized weighed food diary for three days in which they were asked to document meal and snack times, weigh and/or measure the quantity of all foods and drinks, the brand name and serving size consumed, and all BGL measurements. Participants were provided with electronic kitchen scales and measuring cups. Food diaries were analyzed using Foodworks 7 (Xyris, Australia), which uses the Food Standards Australia New Zealand (FSANZ) published AusNut and NUTTAB 2010 databases^21^.

Dietary intake was compared to the Australian National Health and Medical Research Council (NHMRC) age and gender specific nutrient reference values^22^. Dietary intake was compared to the Estimated Average Requirement (EAR), which is a daily nutrient level estimated to meet the requirements of half the healthy individuals in a particular life stage. Inadequate intake in our study is defined as those not meeting EAR recommendations^22^. Energy requirements were calculated by Schofields’ Equation using current weight for the basal metabolic rate multiplied by a physical activity level of 1.2^23^. Sodium and fiber recommendations were adopted from the International Society of Pediatric and Adolescent Diabetes (ISPAD), with adequate fiber calculated as age in years +5 for grams/day^24^. Due to age and gender specific recommendations, proportions meeting the guidelines are reported as opposed to raw values of nutrient intake. Carbohydrate intake documented in the food diary was cross-checked with CGMS reports and insulin pump downloads. Carbohydrates used to treat mild hypoglycemic events (BGL < 4.0 mmol/l) were not included.

### Test meal

All participants were given the same low glycemic index breakfast (Ancient Grains Gluten Free Cereal, Freedom Foods, Australia) for consumption on the first three study days together with portion-controlled ultra-high temperature (UHT) milk (Devondale, Our Lightest One). At the study visit, participants indicated their usual portion of cereal; using this information the dietitian weighed out the same quantity and labeled the carbohydrate amount for each individual breakfast. Participants were asked to refrain from consuming other foods for breakfast.

### CD Status and Gluten Free Diet Adherence

Tissue Transglutaminase (TTG) IgA and deamidated IgG were measured in all participants at least once within the previous 12 months and negative CD serology was confirmed for the T1D only controls. For T1D+CD patients, GFD adherence was assessed both clinically and serologically as previously described^25^. This included documentation of families’ usual dietary intake, including brands of products, family cooking and meal preparation practices, and precautions taken when eating out. GFD adherence was classified by TTG titers in the normal range (or declining titers if recently diagnosed) and assessed as GFD adherent (GFD+) by the dietitian.

### Statistical analysis

Descriptive statistics are reported as mean + standard deviation (SD) for normally distributed data or median (range) for skewed data. Continuous variables, including clinical characteristics and glucose variability parameters, were compared between groups using student’s t-test for normally distributed data and Mann-Whitney *U* tests for skewed data. Categorical data were compared using chi-squared tests. Statistical analyses were performed using SPSS (IBM, SPSS Inc., Chicago, IL, USA) and Stata 14 (Stata Corp., College Station, TX, USA). *P*-values < 0.05 were considered significant.

## Results

Twenty youth (10 with T1D, 10 with T1D+CD) were recruited into the study. Three patients had CGMS equipment failure (two had no data recorded and one had the sensor fall out on the first day of the study). Comparing patients with CD (n = 10) and those without (n = 7), there were no statistically significant differences in demographic or clinical features other than TTG titers (Table 1). Insulin to carbohydrate ratios and insulin sensitivity factors were not different between the two groups, nor percentage of basal insulin. All but one of the T1D+CD patients was GFD adherent, and as results were not significantly different when this patient was excluded, their data were included in the analyses.

### Dietary Intake

Dietary intake per participant was calculated as the average of the three recorded days. Total energy and proportional macronutrient intake were not statistically different between those on the gluten-containing or GFD diet (Table 2). For the micronutrients, more T1D+CD participants met the average daily recommended vitamin C intake from fruit (100 vs 43%, p = 0.006). All together, the majority had inadequate dietary calcium (76%), folate (71%), and fiber (53%) with excessive saturated fat (12% total energy intake) and sodium (>2,000 mg/day) intakes.

### CGMS Data analysis

A total of 2,245 hours of data were recorded by CGMS, with a mean of 132 ± 26 hours and 1,585 ± 314 sensor readings per patient. Blood glucose profiles did not significantly differ between youth with T1D vs T1D+CD (Table 3), including daily mean BGL and times within the hyperglycemic, euglycemic and hypoglycemic ranges. Overall, total, within day and between day variability was not different between groups.

### Meal analysis with CGMS

**Figure 1:**
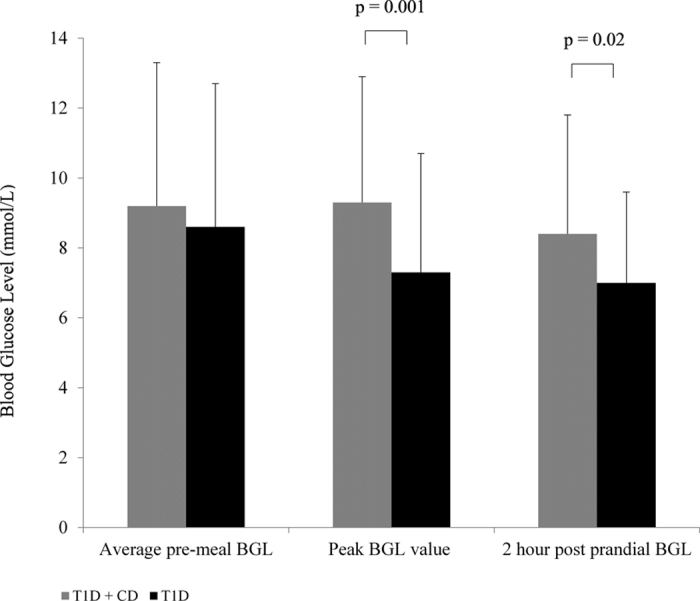
Average pre-meal, peak and post prandial blood glucose levels for meals accompanied with an insulin bolus for youth with T1D+CD compared to T1D alone.

A total of 222 main meals were identified from CGMS traces and pre-meal BGL records throughout the study period (median 16, range 8–18 meals per patient) and of these 179 (81%) were accompanied by an insulin bolus, with no difference in the proportion of delivered boluses in those with or without coexisting CD (81% vs 80%, p = 0.70). Glycemic profiles for meals where an insulin bolus was delivered are reported in Table 3. Youth with T1D+CD experienced faster time to peak BGL (77 ± 32 mins vs 89 ± 34 mins, *P* = 0.03), higher peak BGL values post prandial (9.3 ± 3.6 vs 7.3 ± 3.4, *P* = 0.001), and 2 hour post prandial BGLs (8.4 ± 3.4 vs 7.0 ± 2.6, *P* = 0.02) (Fig. 1). For the test breakfast, youth with T1D+CD had higher peak (11.3 ± 2.5 vs 6.8 ± 4.2, *P* = 0.02) and 2 hour post prandial BGLS (9.2 ± 2.7 vs 5.9 ± 3.1, *P* = 0.02). The difference between post-meal BGL and peak BGL post-meal was significantly correlated with longer CD duration (R = 0.53, *P* = 0.01). In the diabetes alone group, diabetes duration was not associated with change in pre- to peak BGL values (*P* = 0.50).

## Conclusions

This is the first study to examine the impact of the GFD in youth with coexisting CD and T1D on glycemic variability and nutrient intake. The GFD was associated with greater glycemic excursions, characterized by a faster time to peak BGL, higher peak and higher two-hour post prandial BGLs, despite similar exogenous insulin requirements. The GFD and the gluten-containing diet had similar macronutrient distributions that met national nutritional guidelines, however the intake of saturated fat and sodium were above national and international recommendations, while the intake of dietary fiber and calcium was inadequate.

The test meal of GF cereal consumed by all study participants was associated with greater glycemic excursions in youth with T1D+CD compared with their T1D only peers. The observation of faster glucose absorption in those with CD expands on a physiological study in which solutions of increasing glucose concentrations were infused into the small intestine and glucose absorption measured by the production of electrical activity (Apparent Km)^26^. Interestingly, higher apparent Km was correlated with longer duration of GFD consumption in patients with CD and was higher than in controls. Similarly, we found a positive relationship between longer CD duration and higher 2 hour postprandial BGL. This suggests that chronic exposure to the GFD, which has a higher glycemic index, modifies glucose transport and results in more rapid glucose absorption. In support of this hypothesis, three molecules that transport glucose (SGLT1, PEPT1 and NHE3) were higher in patients with treated vs untreated CD^27^, implying that carbohydrates specific to the GFD may alter intra-intestinal gene transcription.

The dietary intake of youth with T1D or T1D+CD was at the lower end of both national and international guideline recommendations for carbohydrates, inadequate for fiber and calcium and high for saturated fats^22^,^24^. The latter finding is consistent with dietary data in youth with T1D from the US and Europe^28^,^29^,^30^,^31^, while the inadequate fiber intake is consistent with data in T1D or CD populations^4^,^10^, but not coexisting T1D and CD. Whilst fiber intake was inadequate for those on the GFD, they consumed more fruit, which is a naturally GF carbohydrate food, and may have offset the expected reduction in carbohydrate grain-based products that is often seen in CD alone studies^10^, but not observed in our study group. The detailed dietary history obtained from all patients in this study, irrespective of coexisting CD, indicate there is scope for improvement in dietary quality and reinforces the importance of dietary education in their management.

The micronutrient intake in our two study groups was comparable, however more than half of youth reported below average daily recommended intakes for calcium, iron and folate. The low intake of folate is surprising, since fortification of wheat flour with thiamin and folic acid is mandatory in many countries across the world including Australia^32^. Further voluntary fortification is strictly regulated, but nonetheless allows limited quantities of other vitamins and minerals such as niacin, riboflavin, calcium and iron to be added to wheat-based breads and cereals^33^,^34^. However there is no mandated fortification of GF foods, which may explain the inadequate intake of folic acid, and iron observed in the GFD in other studies^5^,^33^. Similarly, there was a trend towards more patients with T1D+CD vs T1D not meeting adequate intake of folate (20% vs 43%) and iron (30% vs 57%), but this did not reach statistical significance, which may be due to the small sample size.

Limitations of our study include the small patient numbers, however most CGMS studies have included similar patient numbers and are adequately powered to investigate glucose variability data^20^,^35^. Previous studies of the impact of mixed meals on glucose variability were performed under controlled conditions with supervised meals and boluses, only administering insulin for the carbohydrate content of the meals with no additional correction doses for elevated BGLs administered. In contrast, our free-living study enables a more accurate depiction of life with type 1 diabetes, with correction doses of insulin administered pre-meal, thereby providing an unbiased assessment of post-meal glucose excursions. Whilst 3-day food records are commonly used in practice for assessing nutrient intake^36^, their duration may not be of sufficient length to measure adequate intake for some vitamins and minerals^37^. All but one of our patients adhered to the GFD and therefore our results may not be applicable to those not adherent; this is a population that warrants further study given we and others have demonstrated they have worse glycemic control and are at greater risk of microvascular complications^25^,^38^.

In conclusion, we have demonstrated that youth with T1D+CD have greater post-prandial glucose excursions, suggesting that either the GFD or intestinal characteristics influence glucose absorption. Youth with co-existing T1D and CD have similar macronutrient intake to their peers with T1D alone. Dietary advice for youth with T1D should emphasize increasing fiber, folate and calcium intake, and reducing saturated fat and sodium intake, with further attention to increase thiamin intake for those on the GFD.

## Additional Information

**How to cite this article:** Pham-Short, A. *et al*. Greater postprandial glucose excursions and inadequate nutrient intake in youth with type 1 diabetes and celiac disease. *Sci. Rep.* **7**, 45286; doi: 10.1038/srep45286 (2017).

**Publisher's note:** Springer Nature remains neutral with regard to jurisdictional claims in published maps and institutional affiliations.

## Figures and Tables

**Table 1:** Clinical characteristics of patients with T1D+CD compared with T1D.

	T1D+CD	T1D	*P*-value
Number	10	7	
Age at visit (years)	14.3 ± 3.6	14.7 ± 2.8	0.76
Diabetes duration (years)	6.7 ± 4.0	5.9 ± 2.2	0.63
Celiac Disease duration (years)	3.6 ± 2.7	—	
Anti-deamidated gliadin IgG (ref 0–30)	14 ± 6	7 ± 7	0.25
Anti-tissue transglutaminase IgA (ref 0–30)	22 ± 14	6 ± 3	0.005
HbA1c at visit (%)	7.5 ± 0.7	8.0 ± 1.2	0.34
HbA1c at visit (mmol/mol)	58 ± 3	64 ± 8	0.34
Insulin pump therapy	7 (70%)	7 (100%)	0.11
Total daily dose	44.5 ± 20.0	50.3 ± 17.1	0.57
Insulin units/kg/day	0.81 ± 0.17	0.77 ± 0.12	0.60
% basal insulin	54 ± 4.3	54 ± 9.0	0.88
Insulin to carbohydrate ratio (ICR) (breakfast)	11.1 ± 6.3	10.4 ± 7.0	0.84
Insulin sensitivity factor (ISF) (breakfast)	3.3 ± 2.2	2.7 ± 1.7	0.54
Height SDS	−0.01 ± 1.6	0.8 ± 1.5	0.33
Weight SDS	0.6 ± 0.8	0.9 ± 1.0	0.52
BMI SDS	0.7 ± 0.7	0.9 ± 1.1	0.68
Serum 25 (OH) D (nmol/L) (ref: 51–250)	86 ± 30	72 ± 19	0.24

**Table 2:** Daily dietary analysis for patients with T1D+CD compared with T1D.

	T1D+CD	T1D	*P*-value
Energy (kJ)	8065 ± 2738	7977 ± 2756	0.92
Estimated Energy requirements	7806 ± 1412	8540 ± 1563	0.10
Carbohydrate (g)	229 ± 80	234 ± 66	0.81
Carbohydrate (% total kJ intake)	49 ± 10	51 ± 9	0.35
Protein (g)	84 ± 38	85 ± 39	0.91
Protein (% kJ total intake)	18 ± 6	18 ± 6	0.77
Total fat (g)	72 ± 37	67 ± 40	0.64
Total fat (% kJ total intake)	32 ± 8	29 ± 9	0.24
Saturated fat (g)	26 ± 14	27 ± 20	0.87
Saturated fat (% kJ total intake)	12 ± 4	13 ± 7	0.64
Sodium (mg)	2287 ± 1229	2287 ± 722	0.99
**Patients meeting daily age/gender specific estimated average nutrient requirements** (**%**)			
Dietary fiber	60%	27%	0.20
Thiamin	50%	86%	0.13
Riboflavin	70%	57%	0.59
Niacin equivalents	100%	100%	0.99
**Vitamin C**	**100%**	**43%**	**0.006**
Total folate	20%	43%	0.31
Sodium	20%	14%	0.76
Iron	30%	57%	0.26
Zinc	60%	57%	0.91
Calcium	30%	14%	0.45

**Table 3:** Results of meal time CGMS in youth with T1D+CD compared with T1D accompanied with an insulin bolus.

	T1D+CD	T1D	*P*-value
*All meals*			
Number of meals	110	69	
Average pre-meal BGL	9.2 ± 4.1	8.6 ± 4.1	0.28
Peak BG value	**9.3 ± 3.6**	**7.3 ± 3.4**	**0.001**
2 hour post prandial BGL	**8.4 ± 3.4**	**7.0 ± 2.6**	**0.02**
Area Under Curve	47.4 ± 30.1	47.9 ± 38.3	0.92
Time to peak (mins)	**77 ± 32**	**89 ± 34**	**0.03**
Average daily BGL	9.2 ± 1.8	8.9 ± 2.4	0.83
% time BGL >7.8 mmol/l	55 ± 19	56 ± 27	0.78
% time BGL < 7.8 and >3.8 mmol/l	40 ± 16	37 ± 21	0.77
% time BGL <3.8 mmol/l	5 ± 5	7 ± 8	0.47
SD_w_ Within day variability	4.5 ± 0.6	3.7 ± 0.9	0.09
MODD 5 days Interday variability	3.2 ± 1.2	3.8 ± 0.9	0.38
Test meal			
Test meal starting BGL	7.4 ± 3.0	6.6 ± 2.3	0.49
2 hour post prandial BGL	**9.2 ± 2.7**	**5.9 ± 3.1**	**0.02**
Peak BG value	**11.3 ± 2.5**	**6.8 ± 4.2**	**0.02**
Difference between start and peak BGL	**3.9 ± 1.4**	**0.2 ± 4.8**	**0.05**
